# Discovery of Novel Diagnostic Biomarkers for Common Pathogenic *Nocardia* Through Pan-Genome and Comparative Genome Analysis, with Preliminary Validation

**DOI:** 10.3390/pathogens14010035

**Published:** 2025-01-06

**Authors:** Chaohong Wang, Xinmiao Jia, Ming Wei, Jun Yan, Qing Sun, Sibo Long, Maike Zheng, Yiheng Shi, Guanglu Jiang, Yan Zhao, Hairong Huang, Xinting Yang, Li Gu, Guirong Wang

**Affiliations:** 1 Department of Clinical Laboratory, Beijing Chest Hospital, Beijing Tuberculosis and Thoracic Tumor Institute, Capital Medical University, Beijing 101100, Chinayanjun17318996055@163.com (J.Y.);; 2 Center for Bioinformatics, National Infrastructures for Translational Medicine, Institute of Clinical Medicine & Peking Union Medical College Hospital, Chinese Academy of Medical Sciences and Peking Union Medical College, Beijing 100730, China; 3 Department of Infectious Diseases and Clinical Microbiology, Beijing Institute of Respiratory Medicine and Beijing Chao-Yang Hospital, Capital Medical University, Beijing 100020, China; 4 National Clinical Laboratory on Tuberculosis, Beijing Key Laboratory for Drug Resistant Tuberculosis Research, Beijing Chest Hospital, Beijing Tuberculosis and Thoracic Tumor Institute, Capital Medical University, Beijing 101100, Chinahuanghairong@tb123.org (H.H.); 5 Tuberculosis Department, Beijing Chest Hospital, Capital Medical University, Beijing 101100, China; yl-14t@163.com

**Keywords:** *Nocardia*, *N. cyriacigeorgica*, diagnostic biomarker, pan-genome analysis, comparative genome analysis

## Abstract

The aim of this study was to reveal diagnostic biomarkers of considerable importance for common pathogenic *Nocardia*, utilizing pan-genomic and comparative genome analysis to accurately characterize clinical *Nocardia* infections. In this study, complete or assembled genome sequences of common pathogenic *Nocardia* and closely related species were obtained from NCBI as discovery and validation sets, respectively. Genome annotation was performed using Prokka software, and pan-genomic analysis and extraction of *Nocardia* core genes were performed using BPGA software. Comparative genome analysis of these core genes with the validation-set gene sequences was then performed using BLAT, with a threshold of 30% amino acid coverage and identity, in order to distinguish specific core genes. Finally, candidate gene-specific primers were designed using Snapgene software and DNA samples were obtained from clinical *Nocardia* strains and closely related species for validation. The analysis identified eighteen core genes specific to *Nocardia* spp., four core genes specific to *N. farcinica*, and forty-six core genes specific to *N. cyriacigeorgica*. After rigorous clinical validation, one gene from *Nocardia* spp. and five genes from *N. cyriacigeorgica* were confirmed to have high specificity and therefore can be used as reliable biomarkers for accurate diagnosis of *Nocardia* infection. This pioneering research reveals diagnostic biomarkers of considerable significance, with the potential to substantially enhance the precise diagnosis of common pathogenic *Nocardia* infections, thereby laying the groundwork for innovative diagnostic methodologies in subsequent studies.

## 1. Introduction

*Nocardia* species are Gram-positive, aerobic, weakly acid-resistant actinomycetes that are widely distributed in natural environments such as soil, freshwater, and dust [1]. *Nocardia* is known to cause nocardiosis, a group of serious opportunistic infections that primarily cause lung and skin infections affecting individuals of varying immune statuses [2,3]. In recent years, there has been a notable increase in the number of reported *Nocardia* infections in both immunocompromised and immunocompetent populations [4], and lack of timely and accurate treatment can lead to worsening clinical symptoms and high mortality [5,6,7]. Therefore, accurate and timely diagnosis of *Nocardia* infections is critical to guide appropriate clinical management and improve patient outcomes [7,8,9].

However, the identification of *Nocardia* strains poses significant challenges due to their phenotypic similarities to other bacterial species, particularly *Non-Tuberculous Mycobacteria* (NTM), *Mycobacterium Tuberculosis* (MTB), and other closely related genera [10,11]. These species often share nearly identical clinical symptoms, imaging features, and biochemical parameters, making them difficult to distinguish using traditional identification methods alone [12]. There is also a great deal of variation within *Nocardia* species, and experimenter experience is required to improve detection capabilities. The gold standard for identifying *Nocardia* species is molecular biology, which involves amplification and sequencing of one or two genes, such as *16S rRNA*, *hsp65*, *secA1*, or *sodA* [13,14,15]. However, the highly conserved sequences in these genes limit identification to the species level and may result in misclassification of closely related species [16]. This limitation may be attributed, in part, to the restricted availability of sequencing data, thereby presenting obstacles to achieving accurate strain identification. At present, molecular diagnostic technologies, such as *16S rRNA* PCR and metagenomic next-generation sequencing (mNGS), play a significant role in enhancing the accuracy and speed of pathogen detection. For instance, Wang et al. developed a real-time PCR assay for detecting *Nocardia* in sputum and bronchoalveolar lavage fluid samples [17]. Ding et al. utilized PCR and mNGS to detect *Nocardia* [18]. Despite the advancements made with these technologies, there are still limitations, such as higher costs, and insufficient support for rapid clinical decision-making.

To address this issue, we investigated new diagnostic biomarkers that are specific to common pathogenic *Nocardia* strains. Our goal is to improve the identification of *Nocardia* infections in clinical settings. Firstly, a summary of commonly isolated *Nocardia* species was conducted: *N. farcinica* is prevalent in China, South Africa, Belgium, and France [3,19,20,21]; *N. cyriacigeorgica* is common in Spain and Iran [22,23]; and *N. nova* has the highest isolation rates in the United States and Australia [24,25]. Based on this valuable insight, our comprehensive study focused on these common species. The objective of this study is to identify a set of core genes specific to *Nocardia* through pan-genome and comparative genome analyses that cover the genus *Nocardia* and closely related genera. These core genes may serve as genetic markers to distinguish *Nocardia* strains from other closely related species. The research involved downloading complete or assembled genome sequences of common pathogenic *Nocardia* strains and closely related species from the National Center for Biotechnology Information (NCBI) database (https://www.ncbi.nlm.nih.gov/). A pan-genome analysis was conducted to identify core genes shared among *Nocardia* strains. A 30% amino acid homology threshold was used, followed by further comparative genome analysis to explore unique core genes specific to the genus *Nocardia*.

We identified eighteen *Nocardia*-specific core genes, four *N. farcinica*-specific core genes, and forty-six *N. cyriacigeorgica* specific core genes through the aforementioned comprehensive approach. These specific core genes may have some clinical potential in the diagnosis of *Nocardia* infections. In particular, the specificity of one *Nocardia* spp. gene (*WP_167490169.1*) and five *N. cyriacigeorgica* genes (*WP_130917102.1*, *WP_130916752.1*, *WP_165449001.1*, *WP_014350536.1*, and *GenBank: AVH22254.1*) have been partially validated in clinical samples. In addition, these specific core genes hold promise as the bases for the development of rapid and reliable molecular diagnostic reagents, paving the way for further advances in *Nocardia* diagnostics and therapeutics in the future. This study also provides insight into the accurate identification of different genetically similar pathogens through pan-genomic and comparative genomic analyses.

## 2. Materials and Methods

### 2.1. Genome Sequence Retrieval

We conducted a comprehensive search on the NCBI database to procure the complete or assembled genome sequences of prevalent pathogenic *Nocardia* species and closely related strains, up to August 2022. Our curated genome data collection formed the basis of our investigation. Strains with intact genome sequences were carefully curated and assigned to the discovery set, while strains with assembled genome sequences were allocated to the validation set. As a comparative benchmark, we also included closely related species such as NTM, MTB, *Gordonia*, and *Tsukamurella* in our analysis. The discovery set encompassed a diverse array of 33 *Nocardia* strains, embracing notable pathogens like *N. farcinica* (4 strains), *N. cyriacigeorgica* (3 strains), and *N. brasiliensis* (3 strains), among others. Additionally, it contained 31 strains of NTM, 23 strains of MTB, 12 strains of *Gordonia*, and 7 strains of *Tsukamurella*, each displaying complete genome sequences. Furthermore, the validation set included an extensive compilation of 297 *Nocardia* strains, comprising 40 strains of *N. farcinica*, 32 strains of *N. cyriacigeorgica*, 7 strains of *N. brasiliensis*, and numerous others. It also comprised 108 strains of NTM, 39 strains of MTB, 29 strains of *Gordonia*, and 23 strains of *Tsukamurella*, all accompanied by assembled genome sequences.

### 2.2. Average Nucleotide Identity (ANI), Genome Annotation, and Gene Homology Analysis

To investigate the genomic relatedness between pairs of genomes, we employed Pyani 0.2.10 (https://github.com/widdowquinn/pyani, accessed on 23 December 2023) [26], along with the ANIb algorithm, to calculate the Average Nucleotide Identity (ANI). The genome annotation of all downloaded genome sequences was performed using Prokka 1.14.6 [27], a rapid and efficient prokaryotic genome annotation software. Prokka accurately identified gene and protein sequences, enabling the prediction of potential functions and features for each of these sequences. Furthermore, we utilized OrthoFinder-2.5.5 software [28] to thoroughly examine gene homology across multiple genomes, resulting in the identification of orthologous gene families.

### 2.3. Pan-Genomic and Comparative Genome Analysis: Searching for Nocardia-Specific Core Genes

Initially, the discovery set underwent a pan-genomic analysis that utilized the BPGA-1.3.0 software [29], which allowed us to identify the core genes shared by *Nocardia*. Subsequently, these core genes were subjected to comparative genomic analysis, alongside the gene sequences of the validation set, using BLAT [30]. To ensure robustness, we applied stringent criteria, including 30% coverage and identity thresholds at the amino acid level, to identify *Nocardia*-specific core genes [31].

### 2.4. Preliminary Validation of Specificity Candidate Markers

To validate the clinical applicability of the identified *Nocardia*-specific core genes, we collected clinical samples from both *Nocardia* strains and close relatives identified by *16S rRNA* and *hsp65* gene sequencing [32,33]. Genomic DNA extraction was carried out using the Qiagen DNA Extraction Kit (Dusseldorf, Germany), following the manufacturer’s protocols. Using the specific core-gene sequences as a basis, we conducted primer design utilizing the Snapgene software (version 8.0). For PCR, a reaction mixture (25 μL) containing 12.5 μL of 2*Taq Master Mix (Beijing Kangwei Century Biotechnology Co., Ltd., Beijing, China), 8 μL of nuclease-free water, 2 μL of DNA supernatant, 1 μL of upstream primer, and 1 μL of downstream primer was used. PCR was performed for 35 cycles under the following conditions: denaturation (94 °C for 30 s), annealing (55–59 °C for 30 s), extension (72 °C for 30 s), and a final extension at 72 °C for 2 min. The resulting products were then subjected to agarose gel electrophoresis, with the observed outcomes analyzed under ultraviolet light. By following rigorous experimental procedures, we aimed to ascertain the reliability and specificity of the *Nocardia*-specific core genes for potential clinical applications.

## 3. Results

### 3.1. Genomic Characterization of Common Pathogenic Nocardia Species

The present study encompassed a comprehensive genomic characterization of various pathogenic *Nocardia* species, incorporating 33 complete genomes or genomes assembled at the chromosome level. Among these, common species such as *N. farcinica* (four strains), *N. cyriacigeorgica* (three strains), *N. brasiliensis* (three strains), and others were analyzed. Furthermore, we also included 73 complete genomes, or genomes assembled at the chromosome level, of closely related strains in our analysis, as in the case of NTM, MTB, *Gordonia*, and *Tsukamurella* (Table S1). As for the genomic features, as depicted in Table 1, *Nocardia* had a larger genome (6.2~10.01 Mb) and a higher number of genes (5268~8560) compared with NTM (~6.99 Mb, ~6791), MTB (~4.54 Mb, ~3786), *Gordonia* (~5.96 Mb, ~5191), and *Tsukamurella* (~4.92 Mb, ~6791). Among the *Nocardia* species, *N. farcinica* and *N. cyriacigeorgica*, the two most common pathogenic species, exhibited slightly smaller genome sizes and numbers of genes than the other *Nocardia* species (~6.61 Mb, ~6025; ~6.48 Mb, ~5633). Additionally, the GC content of *Nocardia* (66.5~71.5%) was generally higher than those of NTM (~69%), MTB (~65.5%), and *Gordonia* (~69%), and slightly lower than that of *Tsukamurella* (68~71%).

**Figure 1:**
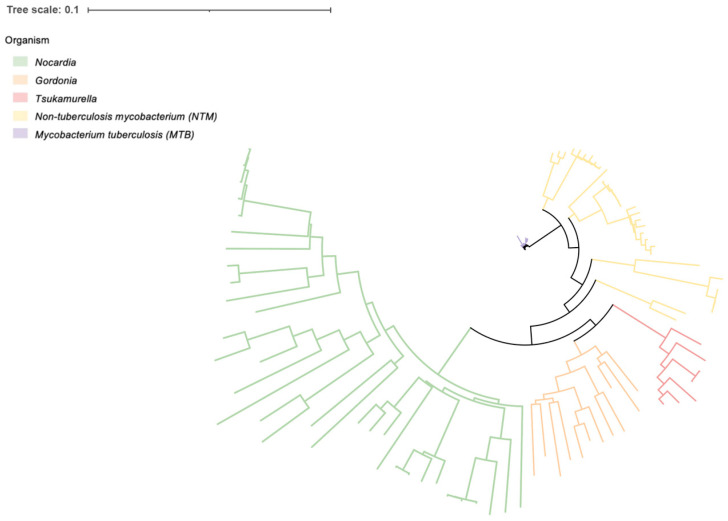
Phylogenetic analyses of *Nocardia* (33), NTM (31), MTB (23), *Gordonia* (12), and *Tsukamurella* (7), with complete genome sequences. Different species are shown in different colors. Systematic Phylogenetic Tree generated using iTOL (https://itol.embl.de, accessed on 23 June 2024).

Subsequently, we conducted a phylogenetic analysis encompassing the aforementioned strains, including *Nocardia* (33), MTB (23), NTM (31), *Gordonia* (12), and *Tsukamurella* (7). As illustrated in Figure 1, the results show that different species cluster in different clades. Notably, separate evolutionary branches were observed for different *Nocardia* species. Particularly, *N. farcinica* and *N. cyriacigeorgica* formed a closely related cluster, representing the most common pathogenic species, while *N. brasiliensis* appeared as a distinct branch (Figure S1). Moreover, the long branch-lengths observed in *Nocardia* species imply the presence of substantial genetic variation among the strains, indicating a noteworthy level of genetic diversity within *Nocardia* spp.

These phylogenetic findings were consistent with the results of our comparative genomic analyses. Strains belonging to the same *Nocardia* species demonstrated higher Average Nucleotide Identity (ANI) values and a greater number of homologous genes compared to those from different species (Table S2A–C). ANI analysis revealed that strains within the same *Nocardia* species exhibited ANI values ranging from 94% to 99%. Conversely, the ANI between different *Nocardia* species, such as *N. farcinica*, *N. cyriacigeorgica*, and *N. brasiliensis*, was approximately 79% (Table S2A). In the context of gene homology analysis (Table S2B,C), strains of the same *Nocardia* species displayed the highest homology, with more than 5000 homologous genes and a proportion of over 92% of shared homologous genes. About 3500 homologous genes (65–85% homologous gene ratio) were found among *N. farcinica*, *N. cyriacigeorgica*, and *N. brasiliensis*. When compared with NTM, MTB, *Gordonia*, and *Tsukamurella*, *Nocardia* exhibited higher homology with *Gordonia* and *Tsukamura*, with approximately 2500 homologous genes and a homologous gene ratio of about 55%. However, the homology with MTB was relatively lower, with around 2200 homologous genes and the proportion of homologous genes at approximately 45% (Table S2A–C).

### 3.2. Pan-Genome and Comparative Genome Analyses Reveal Specific Core Genes (SCGs) of the Common Pathogenic Nocardia Species

**Figure 2:**
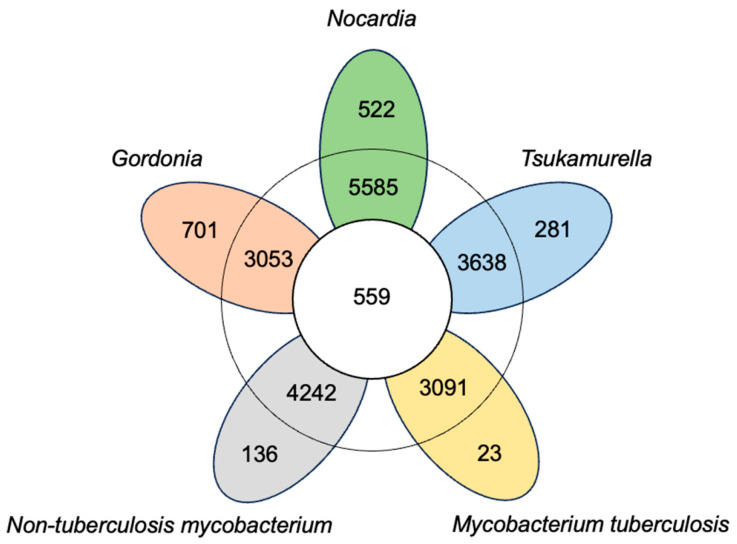
Flower plot showing the core genes, accessory genes, and unique genes of *Nocardia*. The flower plot displays the core-gene numbers (in the center), the accessory gene numbers (in the annulus), and the unique gene numbers (in the petals) of the five organisms.

In order to identify core genes specific to common pathogenic *Nocardia*, we employed a pan-genomic analysis strategy. As controls, we also analyzed closely related strains of NTM, MTB, *Gordonia*, and *Tsukamurella*. The number of core genes among different species was determined through pan-genomic analysis (Figure 2). The pan-gene accumulation curves demonstrated that all species, including *Nocardia*, exhibited an open genome, with the size continuously increasing as more genomes were analyzed (Figure S2). Furthermore, we conducted a pan-genomic analysis of different *Nocardia* strains. There are 6038 orthologous genes in the four *N. farcinica* strains, including 4865 core genes (82.47%); 5573 orthologous genes in the three *N. cyriacigeorgica* strains, including 4369 core genes (77.98%); and 7711 orthologous genes in the three *N. brasiliensis* strains, including 5607 core genes (70.47%) (Table 1). Overall, the percentage of core genes in *Nocardia* species was lower than those observed in NTM, MTB, *Gordonia*, and *Tsukamurella*. Conversely, the percentage of strain-specific genes in *Nocardia* species was higher compared to NTM, MTB, and *Tsukamurella* (Table 1, Figure 2). This difference can be attributed to the conserved genome sequences found in NTM, MTB, *Gordonia*, and *Tsukamurella* strains, in comparison to *Nocardia*.

Subsequently, we conducted a comprehensive comparative genome analysis with two hundred ninety-seven *Nocardia* strains (including forty strains of *N. farcinica*, thirty-two strains of *N. cyriacigeorgica*, seven strains of *N. brasiliensis*, etc.) and one hundred and eight strains of NTM, thirty-nine strains of MTB, twenty-nine strains of *Gordonia*, and twenty-three strains of *Tsukamurella*, the latter set being assembled genome sequences (Table S3), to search for specific core genes that could distinguish common pathogenic *Nocardia* isolates. The numbers of specific core genes exhibited variability among different *Nocardia* species. Eventually, we identified a total of eighteen core genes that were specific to *Nocardia* spp., four core genes that were specific to *N. farcinica*, and forty-six core genes that were specific to *N. cyriacigeorgica*. None were found that were exclusive to *N. brasiliensis*. These specific core genes serve as potential genetic markers that may play crucial roles in distinguishing these common pathogenic *Nocardia* species from one another. Indeed, it is essential to acknowledge that the number of specific core genes may be influenced by the inclusion of different strains in the analysis.

### 3.3. Validation of the Feasibility of Nocardia-Specific Core Genes for Clinical Diagnosis

**Figure 3:**
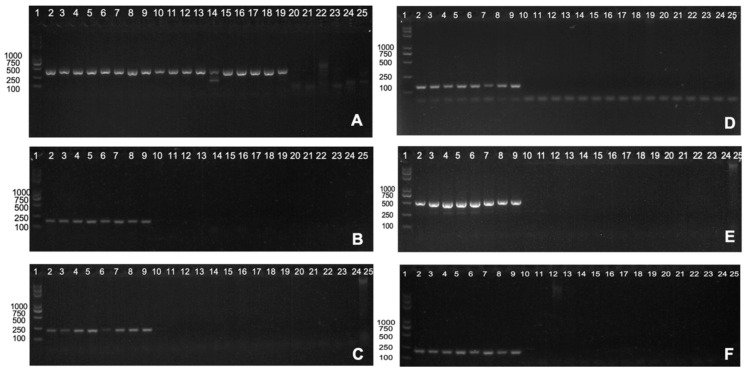
Validation results of clinical samples on the basis of specific core genes for *Nocardia* spp./*N. cyriacigeorgica*. (**A**) Validation results of clinical samples of *Nocardia* spp. based on the *WP_167490169.1* gene (409 bp) (1 Marker; 2–9 *N. farcinica*; 10–13 *N. cyriacigeorgica*; 14 *N. otitidiscaviarum*; 15 *N. abscessus*; 16 *N. wallacei*; 17 *N. beijingensis*; 18 *N. nova*; 19 *N. puris*; 20 *Tsukamurella*; 21 *Gordonia*; 22 *Mycobacterium tuberculosis*; 23–25 *Nontuberculous mycobacteria*). (**B**–**F**) Validation results of clinical samples of *N. cyriacigeorgica* based on the *WP_130917102.1*/*WP_130916752.1*/*WP_165449001.1*/*WP_014350536.1*/*GenBank: AVH22254.1* gene (179 bp/206 bp/139 bp/506 bp/191 bp) (1 Marker; 2–9 *N. cyriacigeorgica*; 10–13 *N. farcinica*; 14 *N. otitidiscaviarum*; 15 *N. abscessus*; 16 *N. wallacei*; 17 *N. beijingensis*; 18 *N. nova*; 19 *N. puris*; 20 *Tsukamurella*; 21 *Gordonia*; 22 *Mycobacterium tuberculosis*; 23–25 *Nontuberculous mycobacteria*).

To validate the clinical feasibility of the screened *Nocardia*-specific core genes, we successfully extracted DNA templates from eighteen samples of *Nocardia* species and six samples of closely related strains, which are listed in Table 2. Following PCR amplification and agarose gel electrophoresis, we identified specific gene markers that showed promising clinical potential for diagnosing nocardiosis. Among the screened genes, one *Nocardia* spp. gene (*WP_167490169.1*) and five *N. cyriacigeorgica* genes (*WP_130917102.1*, *WP_130916752.1*, *WP_165449001.1*, *WP_014350536.1*, and *GenBank: AVH22254.1*) demonstrated robust specificity for the diagnosis of nocardiosis; the gene sequences and primer sequences are presented in Table S4. The association of these specific markers with *Nocardia* infection is supported by our experimental data, as depicted in Figure 3. However, it is important to note that while these markers show specificity, their clinical significance has not been fully established. Regrettably, our current experiment did not yield any core genes specific to *N. farcinica*.

## 4. Discussion

In this study, we conducted comprehensive pan-genome and comparative genome analyses to identify specific core genes that have the potential to serve as diagnostic biomarkers for common pathogenic *Nocardia* species. The primary objective of this study was to address the challenge of accurately diagnosing *Nocardia* infections, which exhibit phenotypic similarities with other bacterial species, particularly NTM, MTB, and other closely related genera [10,11]. The results of this study indicate that the specific core genes identified in this research have the potential to significantly enhance the precise identification of clinical *Nocardia* infections, consequently leading to improved patient outcomes. Firstly, we analyzed the genomic characterization of several common pathogenic *Nocardia* species, revealing interesting insights into their genetic diversity and relevance to other bacterial species. The larger genome size and number of genes in *Nocardia*, compared to NTM, MTB, *Gordonia*, and *Tsukamurella*, suggests a higher level of genetic complexity and adaptability in *Nocardia* spp., consistent with previous descriptions [34,35]. Phylogenetic analyses supported these findings, with *N. farcinica* and *N. cyriacigeorgica* forming a densely packed cluster and *N. brasiliensis* acting as a distinct branch, suggesting distinct evolutionary relationships between these common pathogenic species. The higher homology observed within *Nocardia* spp. than seen in other closely related species may be due to the conserved genome sequences shared among *Nocardia* strains. These results highlight the importance of identifying specific genetic markers to accurately distinguish different *Nocardia* species [34].

Second, a specific set of core genes unique to *Nocardia* spp., *N. farcinica*, and *N. cyriacigeorgica* has been identified through pan-genomic and comparative genome analyses. These specific core genes are critical, as they have the potential to serve as diagnostic markers to distinguish common pathogenic *Nocardia*. The identification of eighteen *Nocardia* spp. specific core genes, four *N. farcinica* specific core genes and forty-six *N. cyriacigeorgica* specific core genes provides valuable genetic targets for the development of rapid and reliable molecular diagnostic reagents. Meanwhile, the successful validation of *Nocardia* specific core genes in clinical samples provides confidence in their clinical feasibility. The *Nocardia* spp. specific core gene (*WP_167490169.1*) and *N. cyriacigeorgica* specific core genes (*WP_130917102.1*, *WP_130916752.1*, *WP_165449001.1*, *WP_014350536.1*, and *GenBank: AVH22254.1*) showed strong specificity and were able to detect the pathogen accurately and promptly. In addition, bioinformatics predictions showed that the marker gene *WP_167490169.1* is predicted to encode a transcriptional regulator of the AraC family, which is likely involved in the regulation of gene expression and may be related to biological processes such as metabolic pathways, stress response, and drug resistance; additionally, the marker gene *WP_130917102.1* encodes NADH-flavin reductase, an enzyme that may be involved in bacterial metabolism and may play an important role in energy and redox homeostasis in *Nocardia*; finally, the functions of *WP_130916752.1*, *WP_165449001.1*, *WP_014350536.1*, and *GenBank: AVH22254.1* have not yet been characterized.

The findings of this study have potential implications for the clinical setting, where timely and accurate diagnosis of *Nocardia* infections is critical for appropriate clinical management and improved patient outcomes. The specific core genes identified could serve as a basis for the development of molecular diagnostic tools. However, there are still some limitations that need to be considered. Firstly, some of the common *Nocardia* such as *N. farcinica*, *N. brasiliensis*, *N. otitidiscavarium*, and *N. asteroides* are missing specific core genes after analysis due to the limited number of complete genome sequences in the database. Secondly, the limited number and type of clinical samples suggest a need for a larger scale validation study at a later stage, which would provide stronger evidence of the clinical utility and reliability of these biomarkers. Furthermore, it should be noted that the present study has not yet compared the performance of these molecular markers with existing diagnostic technologies, such as MALDI-TOF. Planned future work will include such comparative analyses, as well as the validation of the diagnostic potential of these molecular markers on a broader range of clinical samples.

## 5. Conclusions

In summary, this study successfully identified diagnostic biomarkers for specific core-gene forms of common pathogenic *Nocardia* strains. These findings have great potential for advancing the accurate diagnosis of *Nocardia* infections, contributing to improved patient outcomes. The identified genes can serve as a basis for the development of molecular diagnostic tools that will allow rapid and accurate identification of *Nocardia* infections. This study opens new opportunities for the development of novel diagnostic methods and targeted therapies for *Nocardia*-associated infections.

## Figures and Tables

**Table 1:** Genomic features of the common pathogenic *Nocardia*.

Organism	Strain Number	Average Genome Size (Mb)	Average Gene Number (CDS)	Average GC Content (%)	Average Core-Gene Number (%)	Average Accessory Gene Number (%)	Average Unique Genes Number (%)	Average Exclusively Absent Genes Number (%)
*Nocardia*	33	7.8 (6.2~10.01)	6672 (5268~8560)	68.5 (66.5~71.5%)	1773 (26.57)	4329 (64.88)	562 (8.42)	8 (0.12)
*N. brasiliensis*	3	8.77 (~9.44)	7957 (~8551)	68 (~68%)	5607 (70.47)	1098 (13.80)	703 (8.83)	549 (6.90)
*N. cyriacigeorgica*	3	6.32 (~6.48)	5603 (~5633)	68 (~68%)	4369 (77.98)	494 (8.82)	493 (8.80)	247 (4.41)
*N. farcinica*	4	6.45 (~6.61)	5899 (~6025)	70.5 (~70.5%)	4865 (82.47)	651 (11.04)	266 (4.51)	117 (1.98)
*Non-tuberculosis mycobacteria (NTM)*	31	5.86 (~6.99)	4935 (~6791)	66.9 (~69%)	1543 (31.27)	3232 (65.49)	156 (3.16)	4 (0.08)
*Mycobacteria tuberculosis (MTB)*	23	4.42 (~4.54)	3646 (~3786)	65.4 (~65.5%)	3245 (89.00)	381 (10.45)	12 (0.33)	8 (0.22)
*Gordonia*	12	4.99 (~5.96)	4326 (~5191)	67 (~69%)	1351 (31.23)	2148 (49.65)	808 (18.68)	19 (0.44)
*Tsukamurella*	7	4.71 (~4.92)	4561 (~4963)	70.5 (68~71%)	2794 (61.26)	1344 (29.47)	326 (7.15)	97 (2.13)

**Table 2:** Strains used for validation in this study.

Strains	Source of Strains ^1^	No. of Strains
*Nocardia cyriacigeorgica*	BCH	8
*Nocardia farcinica*	BCH-NTCL	8
*Nocardia otitidiscaviarum*	BCH	1
*Nocardia abscessus*	BCH	1
*Nocardia wallacei*	BCH	1
*Nocardia beijingensis*	BCH	1
*Nocardia nova*	BCH	1
*Nocardia puris*	BCH-NTCL	1
*Tsukamurella*	BCH-NTCL	1
*Gordonia*	BCH-NTCL	1
*Mycobacterium* *tuberculosis*	H37Rv (BCH-NTCL)	1
*Mycobacterium chelonei*	ATCC35750 (BCH-NTCL)	1
*Mycobacterium abscess*	ATCC19977 (BCH-NTCL)	1
*Mycobacterium avium*	ATCC25291 (BCH-NTCL)	1

^1^ BCH: Beijing Chaoyang Hospital; BCH-NTCL: National Tuberculosis Clinical Laboratory, Beijing Chest Hospital.

## Data Availability

The original contributions presented in the study are included in the article/Supplementary Materials; further inquiries can be directed to the corresponding authors.

## Supplementary Materials

The following supporting information can be downloaded at: https://www.mdpi.com/article/10.3390/pathogens14010035/s1, Table S1: Infomation of strains with complete genome sequences used in this study; Table S2: Pairwise comparison of ANIs, homologous gene numbers and homologous gene ratio among the common pathogenic *Nocardia*; Table S3: Infomation of strains with assembled genome sequences used in this study; Table S4: Locus_tag, sequence and primer of *Nocardia* spp./*N. cyriacigeorgica* special core genes; Figure S1: Phylogenetic analysis of *Nocardia* (33), NTM (31), MTB (23), *Gordonia* (12), and *Tsukamurella* (7) with complete genome sequences. Different species are shown in different colors. Systematic Phylogenetic Tree generated using iTOL (https://itol.embl.de); Figure S2: Gene accumulation curves for the pan-genome. (A) Gene accumulation curves of the pan-genome (orange) and core-genome (purple) of *Nocardia*, NTM, MTB, *Gordonia* and *Tsukamurella*. (B) Gene accumulation curves of the pan-genome (orange) and core-genome (purple) of *N. farcinica*, *N. cyriacigeorgica* and *N. brasiliensis*).
