# Supplementary material for: Discovery of Novel Diagnostic Biomarkers for Common Pathogenic Nocardia Through Pan-Genome and Comparative Genome Analysis, with Preliminary Validation
Source: Pathogens. 2025 Jan 6;14(1):35. doi: 10.3390/pathogens14010035 (PMC11768141; doi:10.3390/pathogens14010035)
Supplement: Supplementary file 1 [file pathogens-14-00035-s001.zip › pathogens-3301513-supplementary figures.pdf]

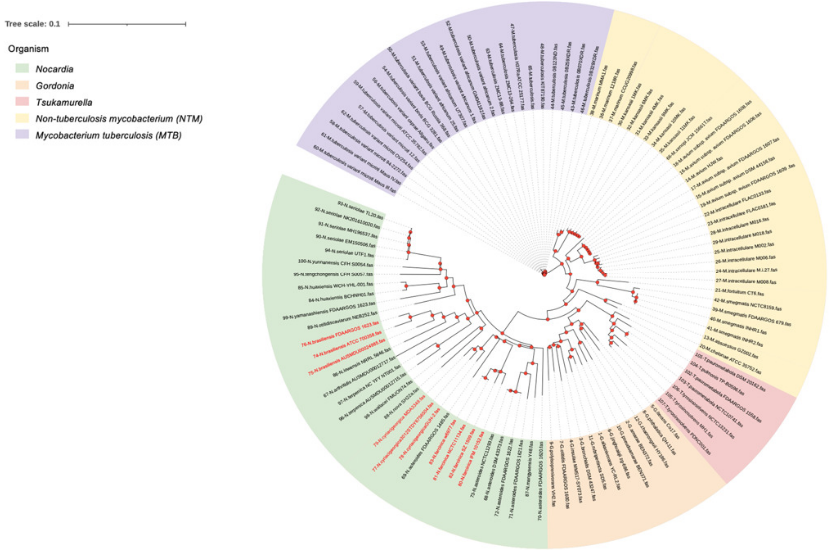

Figure S1. Phylogenetic analysis of *Nocardia* (33), *NTM* (31), *MTB* (23), *Gordonia* (12), and *Tsukamurella* (7) with complete genome sequences. Different species are shown in different colors. Systematic Phylogenetic Tree generated using iTOL (https://itol.embl.de).

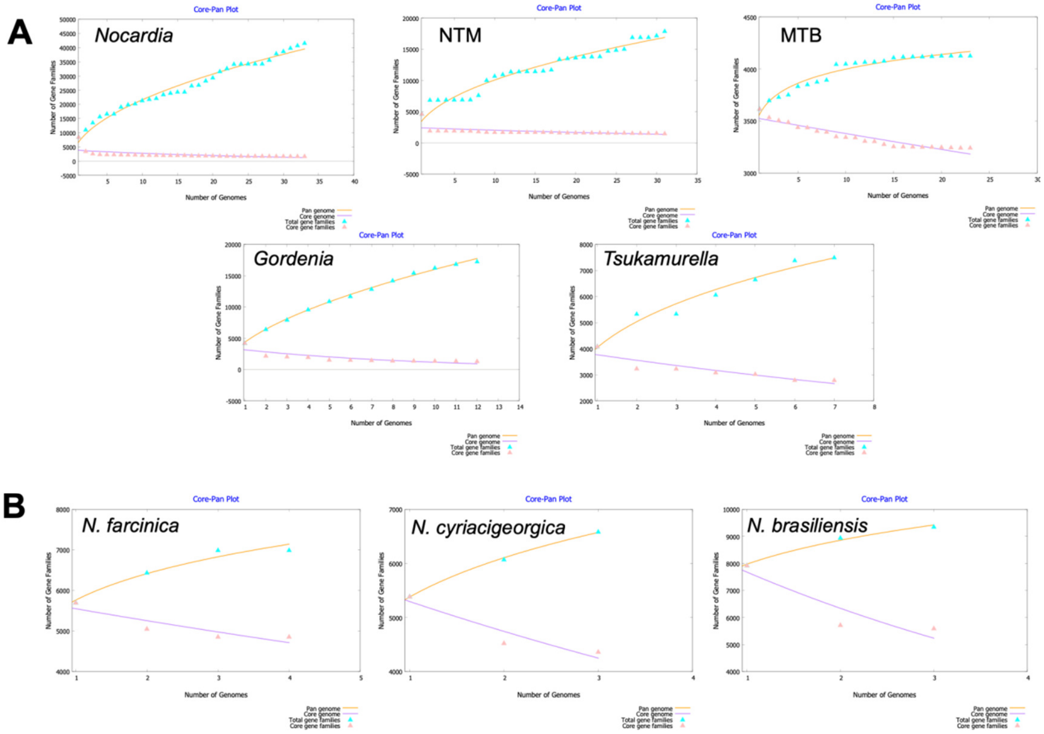

Figure S2. Gene accumulation curves for the pan-genome. (**A**) Gene accumulation curves of the pan-genome (orange) and core-genome (purple) of *Nocardia*, NTM, MTB, *Gordonia* and *Tsukamurella*. (**B**) Gene accumulation curves of the pan-genome (orange) and core-genome (purple) of *N. farcinica*, *N. cyriacigeorgica* and *N. brasiliensis.*
